# The interplay of labile organic carbon, enzyme activities and microbial communities of two forest soils across seasons

**DOI:** 10.1038/s41598-021-84217-6

**Published:** 2021-03-02

**Authors:** Chen-yang Xu, Can Du, Jin-shi Jian, Lin Hou, Zhi-kang Wang, Qiang Wang, Zeng-chao Geng

**Affiliations:** 1 College of Natural Resources and Environment, Northwest A&F University, No. 3 Taicheng Road, Yangling, 712100 Shaanxi China; 2 Key Laboratory of Plant Nutrition and the Agri-environment in Northwest China, Ministry of Agriculture, Northwest A&F University, Yangling, 712100 Shaanxi China; 3 Pacific Northwest National Laboratory-University of Maryland Joint Global Change Research Institute, 5825 University Research Court, Suite 3500, College Park, MD USA; 4 College of Forestry, Northwest A&F University, Yangling, 712100 Shaanxi China

**Keywords:** Biogeochemistry, Ecology, Environmental sciences

## Abstract

Soil labile organic carbon (LOC) responds rapidly to environmental changes and plays an important role in carbon cycle. In this study, the seasonal fluctuations in LOC, the activities of carbon-cycle related enzymes, and the bacterial and fungal communities were analyzed for soils collected from two forests, namely *Betula albosinensis* (Ba) and *Picea asperata* Mast*.* (Pa), in the Qinling Mountains of China. Results revealed that the seasonal average contents of microbial biomass carbon (MBC), easily oxidized organic carbon (EOC), and dissolved organic carbon (DOC) of Pa forest soil were 13.5%, 30.0% and 15.7% less than those in Ba soil. The seasonal average enzyme activities of β-1,4-glucosidase (βG), and β-1,4-xylosidase (βX) of Ba forest soils were 30.0% and 32.3% higher than those of Pa soil while the enzyme activity of cellobiohydrolase (CBH) was 19.7% lower. Furthermore, the relative abundance of *Acidobacteria* was significantly higher in summer than in winter, whereas the relative abundance of *Bacteroidetes* was higher in winter. Regarding the fungal communities, the relative abundance of *Basidiomycota* was lowest in winter, whereas *Ascomycota* predominated in the same season. In addition, the soil LOC was significantly positively correlated with the CBH, βG and βX activities. Changes in LOC were significantly correlated with *Acidobacteria*, *Bacteroidetes* and *Basidiomycota*. We conclude that the seasonal fluctuations in forest soil LOC fractions relied on carbon cycle-associated enzymatic activities and microorganisms, which in turn were affected by climatic conditions.

## Introduction

Soil and vegetation carbon pools in forest ecosystem together contain approximately 1240 Pg of carbon (C)^1^, and soil organic carbon^2^ accounts for 73% of terrestrial soil carbon worldwide^3^. Soil contains more carbon than the sum of atmosphere and terrestrial vegetation^4,5^, thus the impact of soil carbon and climate change cannot be overestimated^6,7^. Soil carbon is generally classified as active carbon, slow carbon, and passive carbon based on turnover time^8,9^. Soil microbial biomass carbon (MBC), easily oxidized organic carbon (EOC), and dissolved organic carbon (DOC) are important indicators of soil labile organic carbon (LOC)^10^. Soil LOC constitutes only a small proportion of the total soil organic carbon (SOC) content, but its decomposition greatly affects the atmospheric CO_2_ concentration^11^. Hence, the atmospheric CO_2_ concentration is highly susceptible to soil LOC fluctuations; as such, fluctuations in LOC may affect global climate change.

Soil microorganisms regulate the transformation of 85–90% of soil organic matter^12^ via various pathways, such as decomposition, polymerization and synthesis. It has been previously shown that the composition of the soil microbial communities is closely associated with SOC changes^13^. Studies showed that soil LOC fractions had positive correlations with the quantities of bacteria and fungi in a mixed forest^14^. Soil enzymes are produced by soil microorganisms and play an important role in the mineralisation of SOC^15^. Cellobiohydrolase (CBH), β-1,4-glucosidase (βG) and β-1,4-xylosidase (βX) regulate the decomposition of organic carbon^16^ and can be used to access microbial metabolism, biogeochemical cycling and microbial nutrient requirements^17^. CBH catalyses the decomposition of cellulose and turns it into fructose and glucose^15^. βG catalyses the final step of cellulose decomposition and further converts unstable cellulose and other carbohydrates into low-molecular-weight compounds^17^. βX mainly catalyses the hydrolysis of xylan into xylooligosaccharides^15^. Soil enzymatic reactions are capable of regulating various biochemical processes, including soil LOC formation and decomposition^18^. However, the effects of microbial communities of forest soils on soil LOC have not been studied^19,20^, and the mechanism of their interactions remains unclear. Understanding their relationships can elucidate the underlying mechanism of SOC transformation.

Soil microbial communities and enzymatic activities are affected by season alternation. On the one hand, season change can affect the types and amount of soil organic matter by regulating the growth and photosynthesis of trees^21^, thereby affecting the soil microbial composition and enzymatic activities. On the other hand, soil microbial communities are also affected by other seasonal climatic factors, such as temperature and humidity^22,23^. A reduction in soil microbial activities at low temperatures is conducive to the accumulation of organic matter. Some studies suggested that changes in soil humidity and temperature were the most likely causes of soil bacterial community variations in temperate forests^21^. Chen et al. found that the enzymatic activities of glucosidase and cellulose peaked in warm seasons due to variations in soil moisture and temperature^19^. However, Qi et al.’s work demonstrated a decline in soil enzymatic activities with increasing temperature in a laboratory incubation experiments, which was attributed to higher decomposition rates of soil organic carbon at higher temperature^20^. Therefore, changes in season and climatic factors are of significant importance, which directly or indirectly affect soil microbial communities and enzymatic activities. However, the controlling factors might differ in different systems; no consistent conclusions have been drawn and thus requiring more investigation.

The Qinling Mountains mark a critical boundary for temperature, rainfall and vegetation of China. Nevertheless, the relationships of soil LOC, enzyme activities, microbial communities across seasons and the controlling factors for such variations are not fully understood for the forest soils in the Qinling Mountains. The objectives of this study were (i) to clarify the seasonal patterns of soil LOC (i.e., MBC, EOC and DOC) in two representative forest soils; (ii) to analyze the relationships among soil LOC, soil enzymatic activities and soil microorganisms; and (iii) to elucidate the main micro-environmental factors affecting soil LOC dynamics.

## Materials and methods

### Site description

Two forests, namely Chinese red birch (*Betula albosinensis*, Ba) and Chinese spruce (*Picea asperata* Mast., Pa), were chosen in the Xinjiashan region, located in the Qinling Mountains of Shaanxi Province, China. The vast majority of Pa and Ba forests are natural secondary forests that were formed after the original forests were felled during the 1960s and 1970s. The Qinling Mountains have a semi-humid continental climate, with an annual mean temperature of 8–10 °C and an annual precipitation of 900–1200 mm, mainly occurring from July to September. The summer at this site is short and warm, whereas the winter is long and cold. The altitude of the Qinling Mountains ranges from 1500 to 2650 m above sea level, and the forest coverage is 96.8%.

### Soil sampling and determination

Soil samples were collected from Pa and Ba forests in July 2015 (summer), October 2015 (autumn), January 2016 (winter) and April 2016 (spring), respectively. Three sampling quadrats (20 m × 20 m) in the same vicinity were selected in each forest. Within each sampling quadrat, 25 sampling points were randomly selected for the collection of top-layer soil (0–10 cm) using a soil auger. The soil samples collected from these 25 sampling points were subsequently mixed into a composite sample. A total of 24 composite soil samples were collected during the four seasons (three samples from two sites in four seasons). Both forest soils in this study were classified as Inceptisol according to the USDA Soil Classification System.

The samples were sieved (< 2 mm) to eliminate large rocks and roots. A portion of each soil sample was immediately transported to the laboratory to determine the soil water content (SWC). The soil subsamples for the molecular analysis were stored on ice, transported to the laboratory and then stored at − 80 °C. The soil subsamples for the MBC and DOC analyses were stored at 4 °C, whereas the other soil subsamples were air-dried and stored at room temperature prior to the SOC, pH, and EOC analyses.

The soil pH was measured by potentiometry (water: soil = 2.5:1)^24^. The SWC was measured using the oven-dry method at 105 ± 2 °C. The SOC content was measured via oxidation by heating with potassium dichromate^25^. The soil temperature (ST) was measured monthly at a 5 cm depth for each sampling point using a pyrometer (Jun 2015 to May 2016). Basic soil properties are presented in Table S1.

MBC was determined using the fumigation–extraction method^26^. EOC was measured via oxidation with KMnO_4_^27^. DOC was measured using the method described by McGill et al.^28^.

The activities of soil carbon cycle-associated enzymes (CBH, βG and βX) were measured via microplate fluorometry based on the fluorescence detection of 4-MUB released from enzymatic hydrolysis^29^. The hydrolysis substrates for these three carbon cycle enzymes are listed in Table 1.

**Table 1:** The enzyme commission numbers and hydrolysis substrates of soil carbon cycle-associated enzymes.

Enzyme	Abbreviation	Enzyme commission numbers	Substrate
Cellobiohydrolase	CBH	3.2.1.91	4-MUB-cellobioside
β-1,4-glucosidase	βG	3.2.1.21	4-MUB-β-d-glucoside
β-1,4-xylosidase	βX	3.2.1.37	4-MUB-β-d-xyloside

4-MUB: 4-methylumbelliferyl.

### Soil microbial communities

#### DNA extraction and quality assessment

Soil DNA was extracted from 0.5 g of fresh soil samples using a FastDNA SPIN Kit (MP Biomedicals, Santa Ana, CA, USA) according to the manufacturer’s instructions. The total quantity of DNA was then assessed using a Thermo NanoDrop 2000 UV Microvolume Spectrophotometer and 1% agarose gel electrophoresis.

#### Primer design and synthesis

The V3-V4 region of 16S rDNA was selected for amplification using the universal forward primer 341F (5′-ACTCCTACGGGAGGCAGCAG-3′) and the reverse primer 806R (5′-GGACTACHVGGGTWTCTAAT-3′)^30^. The ITS2 region of the ITS rDNA hypervariable region was sequenced using the universal forward primer 341F (5′-GCATCGATGAAGAACGCAGC-3′) and the reverse primer 806R (5′-TCCTCCGCTTATTGATATGC-3′)^31^. Specific primers were designed with index and adapter sequences at the 5′ end of the universal primers for MiSeq PE300 sequencing.

#### PCR amplification and Illumina sequencing

The diluted genomic DNA served as templates for PCR amplification using the KAPA HiFi Hotstart ReadyMix PCR Kit (high-fidelity DNA polymerases) for accurate and efficient amplification. Each reaction contained 1 μL of primer, 10 ng of template DNA and 20 μL of PCR System enzyme blend (Roche Applied Sciences, Indianapolis, IN, USA). The PCR conditions for the 16S rRNA gene amplification were initial denaturation for 3 min at 95 °C; 12 cycles of 10 s at 98 °C, 20 s at 72 °C, 20 s at 94 °C, 10 s at 65 °C and 10 s at 72 °C; followed by an extension at 72 °C for 150 s. The PCR conditions for the ITS rDNA gene amplification were initial denaturation for 3 min at 95 °C; 12 cycles of 15 s at 98 °C, 15 s at 72 °C, 10 s at 72 °C, 20 s at 94 °C, 10 s at 38 °C and 10 s at 72 °C; followed by an extension 72 °C for 150 s. The PCR products were separated by 2% agarose gel electrophoresis and recovered from the excised agarose gel using the AxyPrep DNA Gel Extraction Kit. The recovered DNA libraries were then subjected to quality assessment using a Thermo NanoDrop 2000 UV microvolume spectrophotometer and 2% agarose gel electrophoresis and sent to the Illumina MiSeq facility at the Shanghai Realbio Genomics Institute for sequencing.

#### Quality control of the sequencing data

The overlapping read pairs that were generated by paired-end sequencing were merged into single full-length reads of the hypervariable region using Pandaseq software^32^. Subsequently, the merged reads were subjected to the following processing steps using our in-house command script to obtain clean reads: (1) we filtered reads with average quality scores that were less than 20; (2) we filtered reads containing more than 3 N; and (3) we filtered reads beyond the range of 220–500 nt.

#### Bioinformatics analysis

Singletons were filtered from the merged, full-length reads following quality control of the raw data. The sequencing data were subjected to the removal of chimeric reads, and reads with 97% similarity were clustered using Usearch software^33^. Usearch software sorted the reads in order of decreasing abundance to obtain the operational taxonomic units (OTUs), each of which corresponded to a single species^33^. Subsequently, Qiime software was used to construct the rarefaction curve of alpha diversity for the selection of rational randomization parameters^34^. The randomly drawn OTUs were then analysed with Qiime software. One single read was extracted from each OTU as the representative sequence to search against 16S and ITS databases (http://rdp.cme.msu.edu) of known species using the ribosomal database project (RDP) classifier for the species-level classification of each OTU^35,36^. Mothur software (version 1.30.1) was used to calculate the *α*-diversity, including the Chao1 estimator reflecting microbial richness and Shannon index reflecting microbial diversity.

### Statistical analysis

The soil physicochemical properties and enzymatic activities were tested with multifactorial ANOVA (MANOVA) using BM-SPSS 20.0 software. The correlations of soil LOC with SOC and the enzymatic activities of CBH, βG and βX were analysed using Pearson’s correlation coefficients. The correlation of soil LOC with the microbial community composition was analysed via redundancy analysis (RDA) using Canoco 5.0 software.

## Results and discussion

### Seasonal dynamics of soil temperature and LOC component

The monthly mean STs of the Ba and Pa forests from June 2015 to June 2016 are shown in Fig. S1. Continuous monitoring confirmed significant seasonal changes in soil temperature. As shown in Fig. S1, the mean soil temperatures of both forests peaked in July and decreased to below 0 °C from December 2015 to February 2016.

**Figure 1:**
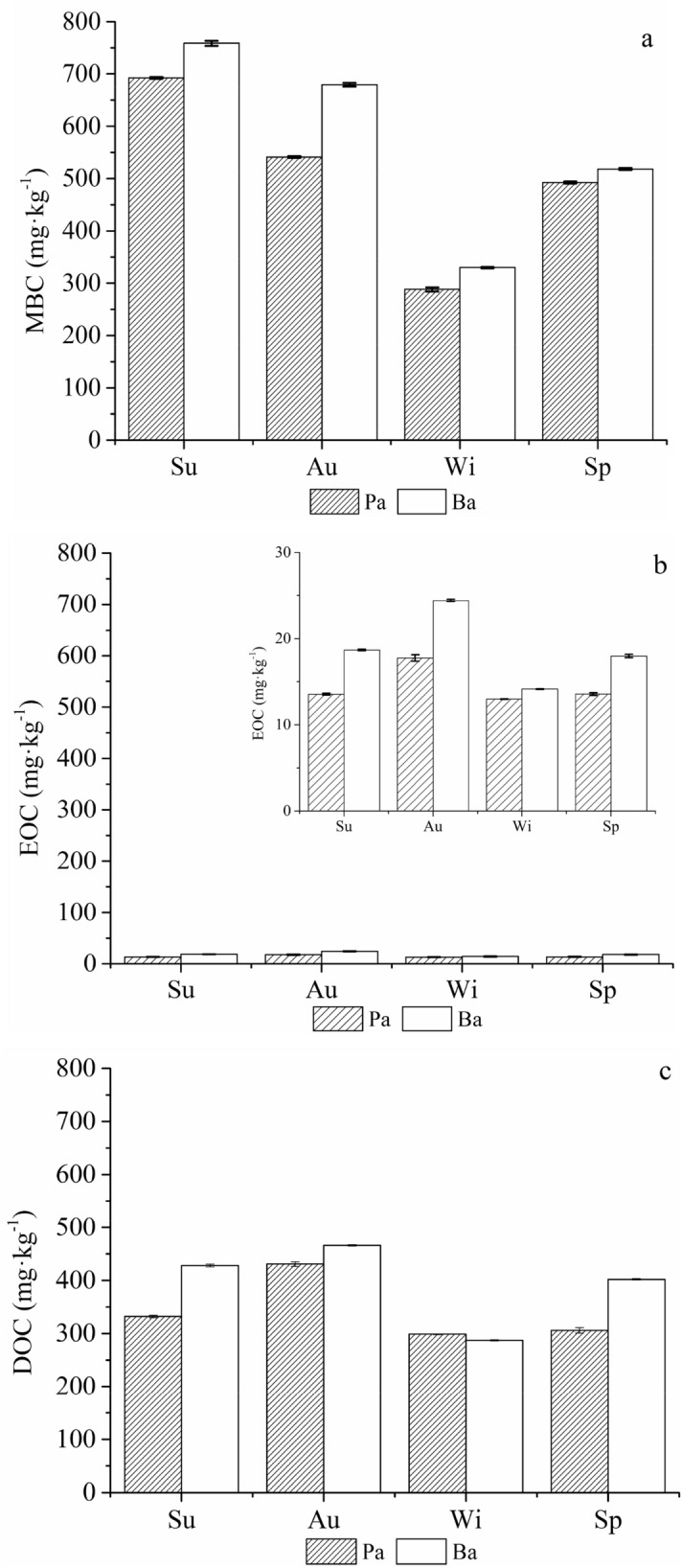
Changes in MBC (**a**), EOC (**b**) and DOC (**c**) in the four seasons. The error bars show standard errors. Sp, Su, Au and Wi represent spring, summer, autumn, and winter, respectively. Pa: *Picea asperata* Mast.; Ba: *Betula albosinensis*; MBC: microbial biomass carbon, EOC: easily oxidized organic carbon; and DOC: dissolved organic carbon (this figure is created by Origin, version 9.0, 2012, OriginLab, Northampton, MA, USA; software available at: https://www.originlab.com/).

Soil MBC, EOC and DOC contents are shown in Fig. 1 while the effects of forest type and season on these three LOC fractions are shown in Table S2. There were significant differences in MBC, EOC and DOC for different forest types and seasons (*p* < 0.01) (Table S2). Forest type and season showed significant interactions with the soil MBC, EOC and DOC contents (*p* < 0.01) (Table S2).

The MBC concentrations in the Pa and Ba soils both reached their highest values in summer (Fig. 1a). The Ba forest had a significantly higher soil MBC content than the Pa forest in all four seasons (*p* < 0.01). The soil MBC content of the Pa forest in summer was 27.9%, 140.2% and 40.6% higher than those in autumn, winter and spring, respectively (*p* < 0.01). The soil MBC content of the Ba forest peaked in summer, which was 11.7%, 130.0% and 46.5% higher than those in autumn, winter and spring, respectively (*p* < 0.01).

The Ba forest had a significantly higher soil EOC content than the Pa forest in all four seasons (*p* < 0.01) (Fig. 1b and Table S2). The soil EOC concentrations in the Pa and Ba soils tended to increase from summer to autumn, then decrease from winter to spring and then increase again from winter to spring.

The Ba forest had a significantly higher soil DOC content than the Pa forest in all four seasons (*p* < 0.01) (Fig. 1c and Table S2). Similar to the EOC content, the soil DOC contents of both forests showed significant seasonal patterns (Fig. 1c). The DOC concentrations in the two forests in autumn were significantly higher than those in summer, winter and spring (*p* < 0.05).

MBC, EOC and DOC are LOC that can serve as early indicators of soil quality because they can regulate the organic matter and nutrient availability in soils and are highly sensitive to environmental changes^37–39^. Our study showed that the Pa forest had a lower soil LOC content than the Ba forest, which is consistent with the findings of most previous studies^40,41^. The LOC in forest ecosystems relies on the types of vegetation. Litterfall and roots are the main sources of organic carbon. Therefore, the litterfall and fine roots of different forest types are the main factors affecting the LOC pools in soils^42,43^. Compared to Pa being evergreen needle leaf forest, Ba of broadleaf forest have relatively greater annual litterfall, fine root biomass, dead fine roots, litterfall exudates, SOC inputs and mineralizable SOC contents, which improve the microbial biomass and activities that serve as additional sources of LOC^44^.

In terms of seasonal fluctuations, the soil LOC content peaked in summer, which contrasts with the results reported by Jiang et al. on early bamboo, *Phyllostachys praecox,* forest soil^45^. They concluded that both MBC and water-soluble organic carbon peaked in winter. Our results were attributable to seasonal fluctuations in ST and SWC. It has been previously indicated that seasonal fluctuations of soil LOC are primarily affected by soil temperature and humidity^46,47^. Soil temperature and humidity affect the soil LOC content by influencing SOC inputs and plant growth^48^. In addition, ST has long been suggested as the key factor regulating the process of litterfall decomposition^49^. Temperature controls the organic matter decomposition rate, resulting in seasonal variations in SOC and microbial metabolism activities. The decomposition of forest litterfall is accelerated by relatively active soil microorganisms in summer^50^ due to higher temperature and precipitation, vigorous plant and microbial growth, accelerated plant photosynthesis and metabolism as well as elevated root exudation^51^, all of which enhance the accumulation, decomposition and transformation of SOC^52^. Subsequently, microbial activities and abundances decreased gradually with decreasing temperature from autumn to winter. Furthermore, the plants largely stopped growing, which resulted in reduced root exudation and attenuated litterfall decomposition, thus lowering the soil LOC content^44^.

### Activities of soil carbon cycle-associated enzymes

There were significant differences in the activities of CBH, βG and βX in different forest types and seasons (*p* < 0.01) (Table S3). There was a significant interaction effect of forest type and season on the activities of CBH, βG and βX (*p* < 0.01) (Table S3).

**Figure 2:**
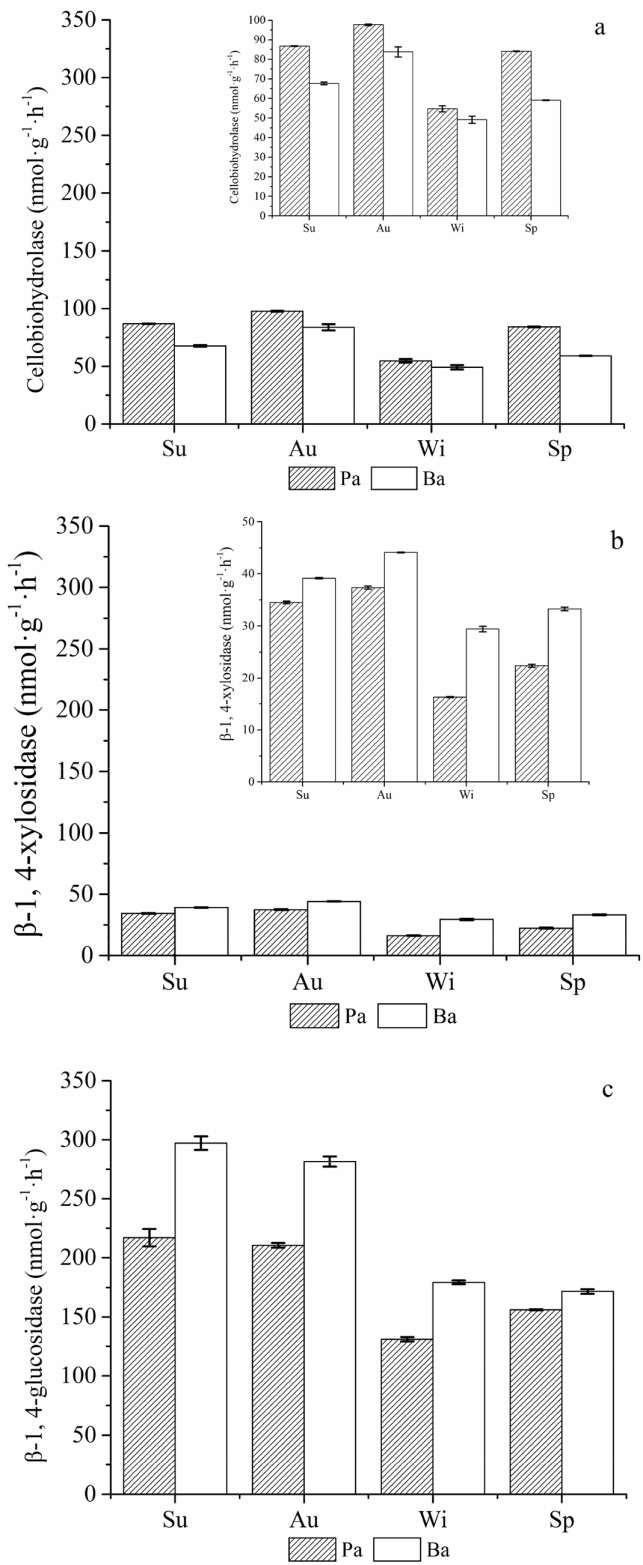
Changes in cellobiohydrolase (**a**), β-1,4-xylosidase (**b**), and β-1,4-glucosidase (**c**) in the four seasons. The error bars show standard errors. Sp, Su, Au and Wi represent spring, summer, autumn, and winter, respectively. Pa: *Picea asperata* Mast.; Ba: *Betula albosinensis* (this figure is made by Origin, version 9.0, 2012, OriginLab, Northampton, MA, USA; software available at: https://www.originlab.com/).

The soil CBH activities in both forests were highest in autumn and reached their lowest levels in winter before increasing again in spring (Fig. 2a). The Pa forest had significantly higher soil CBH activities than the Ba forest (*p* < 0.01) (Fig. 2a and Table S3). The CBH activities in the Pa forest in autumn were significantly higher than those in summer, spring and winter (*p* < 0.01) (Fig. 2a and Table S3). The CBH activities in the Ba forest were consistent with the results observed in the Pa forest.

Among the different vegetation types, the βX activities showed similar seasonal patterns to those of CBH (Fig. 2b). The soil βX activities in both forests had significant seasonal variations (*p* < 0.01) (Fig. 2b and Table S3), among which, the Ba forest had relatively higher soil βX activities in autumn.

The Ba forest had significantly higher soil βG activity than the Pa forest (*p* < 0.01) (Fig. 2c and Table S3). The soil βG activities in the Pa forest were highest and lowest in summer (217.1 nmol g^−1^ h^−1^) and winter (131.1 nmol g^−1^ h^−1^), respectively (Fig. 2c). Moreover, the soil βG activity of the Ba forest in summer was 5.5%, 65.7% and 73.1% higher than those in autumn, winter, and spring respectively (*p* < 0.01) (Fig. 2c and Table S3).

### Seasonal fluctuations in microbial communities

**Figure 3:**
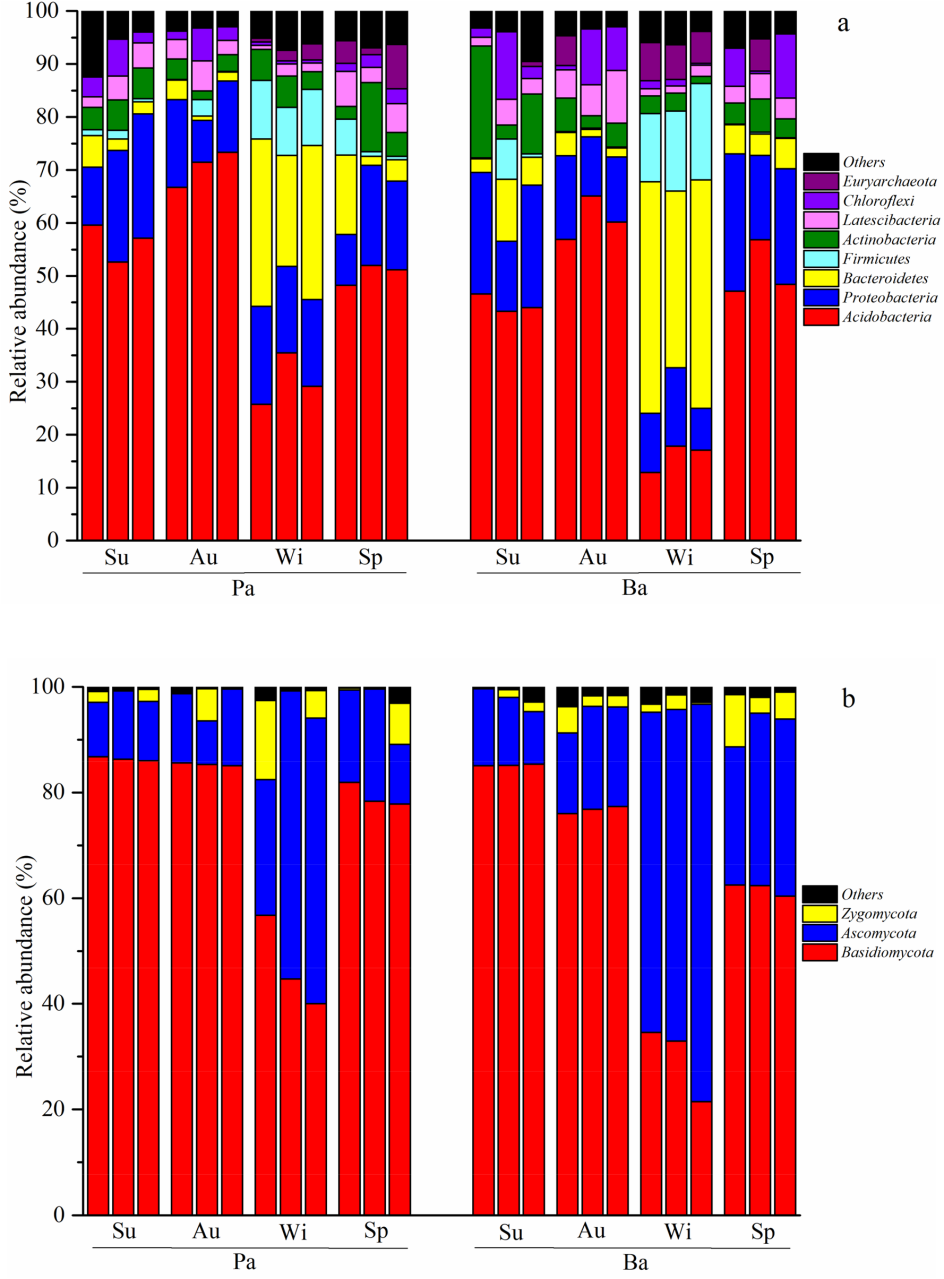
Distribution of the 16S rRNA (**a**) and ITS (**b**) sequences across the bacterial communities. Sp, Su, Au and Wi represent spring, summer, autumn, and winter, respectively (his figure is made by Origin, version 9.0, 2012, OriginLab, Northampton, MA, USA; software available at: https://www.originlab.com/; database).

The HiSeq high-throughput sequencing results indicated that *Acidobacteria*, *Proteobacteria*, *Bacteroidetes*, *Actinobacteria*, *Firmicutes*, *Chloroflexi* and *Latescibacteria* were dominant in both forest soils of the Qinling Mountains, among which *Acidobacteria*, *Proteobacteria* and *Bacteroidetes* constituted up to 70% of the total bacterial abundance (Fig. 3a). The relative abundance of *Acidobacteria* in the Pa forest soil peaked (70.5%) in autumn, which was 24.9%, 134.0% and 46.7% higher than those in summer, winter and spring, respectively. In contrast, the relative abundance of *Bacteroidetes* peaked (27.22%) in winter, which was 681%, 1227% and 273% higher than those in summer, autumn and spring, respectively. Moreover, the relative abundance of *Proteobacteria* peaked in summer. On the other hand, the relative abundance of *Acidobacteria* in the Ba forest soil peaked (60.7%) in autumn, which was 16.5–280.7% higher than those in the other seasons. The relative abundance of *Bacteroidetes* peaked in winter, which was 509–1496% higher than those in the other seasons. In addition, the relative abundances of *Actinobacteria*, *Firmicutes*, *Chloroflexi* and *Latescibacteria* varied seasonally, with abundances ranging from 4.4–11.7%, 0.2–15.4%, 1.0–6.5% and 1.6–7.0%, respectively.

*Basidiomycota* and *Ascomycota* were predominant in both types of forest soils, accounting for more than 90% of the total fungal abundance (Fig. 3b). The relative abundance of *Basidiomycota* in the Pa forest soil peaked (86.4%) in summer, which was 1.2%, 83.1% and 8.8% higher than those in autumn, winter and spring, respectively. However, *Ascomycota* showed an opposite seasonal pattern to that of *Basidiomycota*; the relative abundance in winter was 288.8% higher than that in summer. Moreover, the relative abundance of *Zygomycota* varied seasonally between 1.5 and 6.8%. On the other hand, the relative abundance of *Basidiomycota* in the Ba forest soil peaked in summer, which was 11.0%, 187.1% and 38.0% higher than those in autumn, winter and spring, respectively. The relative abundance of *Ascomycota* in winter was 115.1–430.8% higher than those in spring, summer and autumn, respectively.

Table 2 shows that the Chao1 index of Pa and Ba were both highest in summer while they reached the lowest values in winter. The Shannon index of Pa and Ba was also highest in summer. But the Shannon index of Pa was lowest in winter and that of Ba was lowest in spring.

**Table 2:** Mean α-diversities of the bacterial communities in different seasons.

α-diversity	Forest type	Su	Au	Wi	Sp
Chao 1	Pa	2243.2 ± 38.5a	1919.5 ± 9.9b	752.9 ± 170.4d	1518.2 ± 11.3c
	Ba	2358.7 ± 68.8a	1853.9 ± 29.4b	969.9 ± 24.2d	1422.3 ± 112.7c
Shannon	Pa	8.7 ± 0.1a	8.2 ± 0.0b	7.5 ± 0.0c	7.8 ± 0.0bc
	Ba	8.7 ± 0.0a	7.6 ± 0.2bc	8.2 ± 0.4ab	7.4 ± 0.3c

Different letters stand for significant differences at the 0.05 level. Sp, Su, Au and Wi represent spring, summer, autumn, and winter, respectively. Pa: *Picea asperata* Mast.; Ba: *Betula albosinensis* (data in this table is analyzed by Mothur, version 1.30.1, 2013, University of Michigan, Ann Arbor, MI, USA; software available at: https://mothur.org/).

### Correlations between soil LOC and the carbon cycle enzymatic activities

There were significant positive correlations between MBC and CBH/βG/βX, as well as between DOC and βX and CBH, in the Pa forest soil (*p* < 0.01) (Table 3). EOC was significantly positively correlated with CBH and βX (*p* < 0.05). In addition, there were highly significantly positive correlations between MBC, EOC and DOC with CBH, βG and βX in the Ba forest soil (*p* < 0.01).

**Table 3:** Correlation analysis between soil active organic carbon and enzyme activities.

	CBH	βX	βG
**Pa**			
MBC	0.795**	0.834**	0.891**
EOC	0.693*	0.705*	0.556
DOC	0.725**	0.805**	0.689*
**Ba**			
MBC	0.787**	0.866**	0.888**
EOC	0.967**	0.943**	0.667*
DOC	0.896**	0.912**	0.713**

*Correlation is significant at the 0.05 level, **Correlation is significant at the 0.01 level; Pa: *Picea asperata* Mast.; Ba: *Betula albosinensis*; MBC: microbial biomass carbon, EOC: easily oxidized organic carbon, DOC: dissolved organic carbon; SOC: soil organic carbon; CBH: Cellobiohydrolase, βG: β-1,4-glucosidase, βX: β-1,4-xylosidase (data in this table is analyzed by SPSS, version 20.0, 2011, SPSS Inc., Chicago, IL, USA; software available at: https://www.ibm.com/analytics/spss-statistics-software).

Our results showed that the soil enzymatic activities were significantly affected by seasonal changes and forest type (Table S3), and the results were consistent with other studies^53,54^. These carbon cycle-associated enzymes in both forests had relatively higher activities in summer and autumn and the lowest activities in winter (Fig. 2); the results were consistent with a previous study on soil enzymatic activities of sawtooth oak, *Quercus acutissima,* and Chinese red pine, *Pinus massoniana,* forests^54^. Our results were attributable to seasonal fluctuation in ST, as the relatively high temperature, root exudation, and soil microbial activities facilitated the elevation of enzymatic activities^55,56^.

Microbial enzymes are directly involved in SOC decomposition and synthesis^57^, each with their own substrate and catalytic activity in specific biochemical reactions^58^. Here, we confirmed our hypothesis that the soil LOC contents were significantly correlated with enzymatic activities associated with the carbon cycle. Similar results were also reported by Xiao et al., who found significant correlations between soil LOC and the enzymatic activities of CBH, βG and βX in four typical wetlands^14^. One possible explanation for the significant positive correlations between CBH, βG and βX with the soil MBC, EOC and DOC is that CBH, βG and βX could promote LOC formation^59^. In general, CBH, βG and βX can enhance the mineralization and loss of organic carbon, as these enzymes are involved in the decomposition of cellulose into LOC^18^. The current study indicates that CBH, βG and βX are positive factors stimulating the formation of LOC.

### Correlations between soil LOC and the bacterial and fungal communities

**Figure 4:**
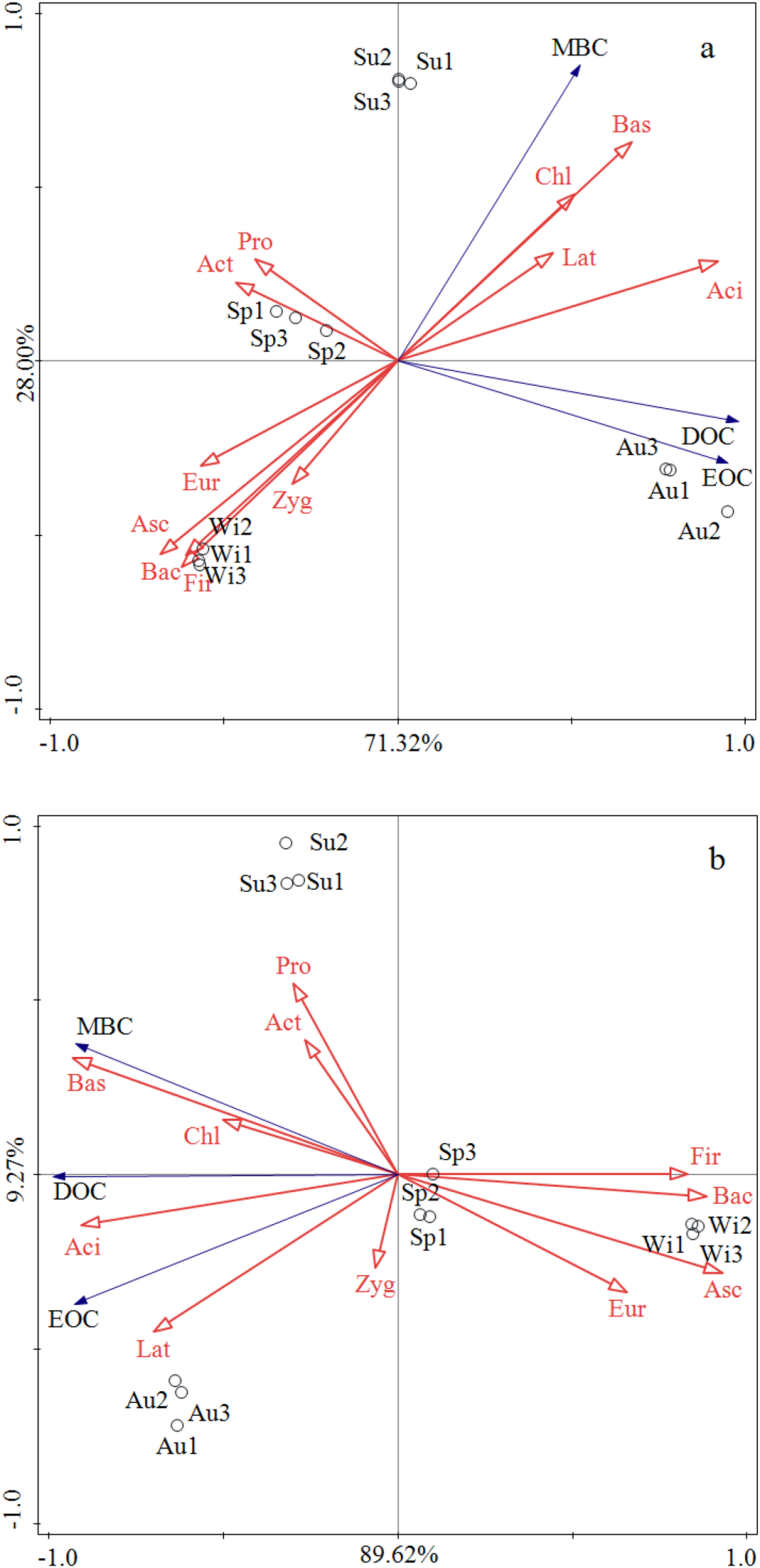
Ordination plots of the redundancy analysis (RDA) to identify the relationship between the abundance of microbial taxa (blue arrows) and LOC (red arrows). (**a**) The relationship between the soil microbial taxa and soil active organic carbon in *Picea asperata* Mast.; (**b**) the relationship between the soil microbial taxa and soil active organic carbon in *Betula albosinensis*. Sp, Su, Au and Wi represent spring, summer, autumn, and winter, respectively. The abbreviations of the microbial taxa are as follows: Aci: *Acidobacteria*; Pro: *Proteobacteria*; Bac: *Bacteroidetes*; Fir: *Firmicutes*; Act: *Actinobacteria*; Lat: *Latescibacteria*; Chl: *Chloroflexi*; Eur: *Euryarchaeota*; Bas: *Basidiomycota*; and Asc: *Ascomycota* (this figure is made by Canoco, version 5.0, 2012, Microcomputer Power, Ithaca, NY, USA; software available at: http://www.canoco5.com/).

The results of RDA indicated that the cumulative explanatory power of the variables on the first axis for the Pa and Ba forests were 71.3% and 89.6%, respectively (Fig. 4). The soil LOC in the Pa forest was primarily affected by *Acidobacteria*, *Basidiomycota*, *Bacteroidetes* and *Firmicutes* (Fig. 4a), with explanatory rates of 62.8% (*F* = 16.9, *p* = 0.001), 43.4% (*F* = 7.7, *p* = 0.005), 41.9% (*F* = 7.2, *p* = 0.008) and 37.2% (*F* = 5.9, *p* = 0.012), respectively. Furthermore, the soil LOC in Ba was primarily affected by *Ascomycota*, *Basidiomycota*, *Bacteroidetes* and *Acidobacteria* (Fig. 4b), with explanatory rates of 78.4% (*F* = 36.2, *p* = 0.001), 36.1% (*F* = 7.7, *p* = 0.001), 66.6% (*F* = 20.0, *p* = 0.003) and 63.5% (*F* = 17.4, *p* = 0.002), respectively.

Figure 4 confirmed our initial hypothesis that changes in soil LOC were significantly associated with bacteria and fungi. Soil microbial communities may regulate various ecological processes, such as litterfall decomposition and mineralization, and lead to dramatic changes in soil carbon dynamics that eventually alter the carbon cycle. The presence of significant positive correlations between soil LOC and bacteria/fungi demonstrated that bacteria and fungi are involved in the formation of labile compounds in SOC.

With regard to bacterial communities, we found that *Acidobacteria* was closely associated with soil LOC (Fig. 4). Soil carbon is mainly affected by bacteria through litterfall decomposition. Previous studies have shown that *Acidobacteria* can grow on media that are supplemented with plant polymers as substrates, indicating that this enzyme plays an important role in the decomposition of plant residues^60^. For example, *Telmatobacter bradus*, 2 *GP1* isolates (KBS83 and CCO287) and *GP3* are *Acidobacteria* that have been proven to be capable of degrading cellulose^61,62^. Eichorst et al. isolated two *Acidobacteria* strains with cellulose-degrading potential from agricultural grassland soils using a medium that was supplemented with complex plant polymers^62^. *Acidobacteria* decompose cellulose mainly via various secretory enzymes. The genomes of some *Acidobacteria*, such as *A. capsulatum*, ‘*Koribacter versatilis*’ Ellin345 and ‘*Solibacter usitatus*’ Ellin6076 strains contain genes encoding β-glucosidases, suggesting that these microorganisms are able to enhance LOC formation by degrading cellulose^63^. The presence of significant positive correlations between *Acidobacteria* and EOC, DOC and MBC in this study indicated that *Acidobacteria* promoted LOC formation via their metabolic processes.

Soil fungi exhibit high decomposing capacities and mainly affect soil LOC by secreting extensive amounts of enzymes that are involved in the decomposition of organic matter, especially refractory organic substances, such as chitin and lignin. Our study indicated that *Basidiomycota* and *Ascomycota* were the dominant fungal species in both types of forests. Both of these fungi promoted carbon transformation^64,65^ by expressing enzymes that are essential for cellulose degradation^66^. *Basidiomycota* mostly rely on exogenous substances as primary carbon sources, such as plant litterfall or soil organic matter, and are involved in the process of soil carbon transformation^45^. *Ascomycota* have an apparent advantage in the metabolism of refractory organic macromolecules, as they can secrete a vast number of enzymes that decompose chitin and lignin^67^. In this study, the EOC and DOC contents were positively correlated with *Basidiomycota* (Fig. 4), indicating that the soil LOC content increased as the relative abundance of *Basidiomycota* increased.

## Conclusions and future prospects

The interplay of soil LOC fractions, bacteria/fungi communities and the activities of carbon-cycle related enzymes in Pa and Ba forest soils of the Qinling mountains were demonstrated. Compared with Ba forest soil, Pa soil was featured with lower contents of SOC, MBC, EOC and lower activities of βG and βX, which were mainly attributable to the differences in litterfall and root exudates of coniferous forest (Pa) and broadleaf forest (Ba). In warm reasons of summer and autumn, the contents of MBC, DOC and the activities of CBH, βG and βX were higher for both Pa and Ba forest soils due to apparently higher ST. In summer, the activities of soil microorganisms were higher due to temperature increase, so were the decomposition rates of organic fractions. In autumn, a large amount of litterfall was input into the soils. More labile organic compounds from rhizodeposition and forest litter became available, thus enhancing the activities of soil organisms and enzymes. Season alternation promoted the shift of microbial communities. Dominant microorganisms changed from *Acidobacteria* and *Basidiomycota* in summer to *Bacteroidetes* and *Ascomycota* in winter. Soil LOC fractions were in significantly positive correlation with the enzyme activities of CBH, βG and βX and the relative abundances of *Acidobacteria* and *Basidiomycota.* Soil LOC and the relative abundance of *Bacteroidetes* were significantly negatively correlated.

Our study revealed that the seasonal dynamics of soil LOC fractions were caused by the variations of microbial communities and carbon-cycle related enzymes. Therefore, the enzyme activities of CBH, βG and βX and the microbial abundances can serve as active biological indicators of forest soil LOC turnover. Season alternation is an important driving force for activities of carbon-cycle related enzymes and shift of microbial communities. The present study indicates that the dynamic interplay among plant, soil and microbial communities is an effective route to deepen our understanding of the forest soil carbon cycling. Future study should also pay attention to the subsurface soil layers due to the apparent vertical stratification of soil physicochemical properties. Furthermore, making an effort to isolate functional bacteria related to carbon-cycle can be rewarding in exploring the underlaying mechanisms of SOC synthesis and decomposition.

## Supplementary Information

Supplementary Information.
