# Supplementary material for: The interplay of labile organic carbon, enzyme activities and microbial communities of two forest soils across seasons
Source: Sci Rep. 2021 Mar 2;11:5002. doi: 10.1038/s41598-021-84217-6 (PMC7925553; doi:10.1038/s41598-021-84217-6)
Supplement: Supplementary file 1 — Supplementary Information. [file 41598_2021_84217_MOESM1_ESM.docx]

Electronic Supplementary Information

**The interplay of labile organic carbon, enzyme activities and microbial communities of two forest soils across seasons**

Chen-yang Xu ^a,b†^, Can Du ^a,b†^, Jin-shi Jian ^c^, Lin Hou ^d^, Zhi-kang Wang ^a,b^, Qiang Wang ^a,b^, Zeng-chao Geng ^a,b*^

^a^ College of Natural Resources and Environment, Northwest A&F University, Yangling, Shaanxi 712100, China

^b^ Key Laboratory of Plant Nutrition and the Agri-environment in Northwest China, Ministry of Agriculture, Northwest A&F University, Yangling, Shaanxi 712100, China

^c^ Pacific Northwest National Laboratory-University of Maryland Joint Global Change Research Institute, 5825 University Research Court, Suite 3500, College Park, MD, United States

^d^ College of Forestry, Northwest A&F University, Yangling, Shaanxi 712100, China

^†^Chen-yang Xu and Can Du contribute equally to this work.

***Correspondence:** Zeng-chao Geng

E-mail: [gengzengchao@126.com](mailto:gengzengchao@126.com).

Tel.: +86-029-87011863;

Address: College of Natural Resources and Environment, Northwest A&F University, No. 3 Taicheng Road, Yangling, Shaanxi 712100, China.


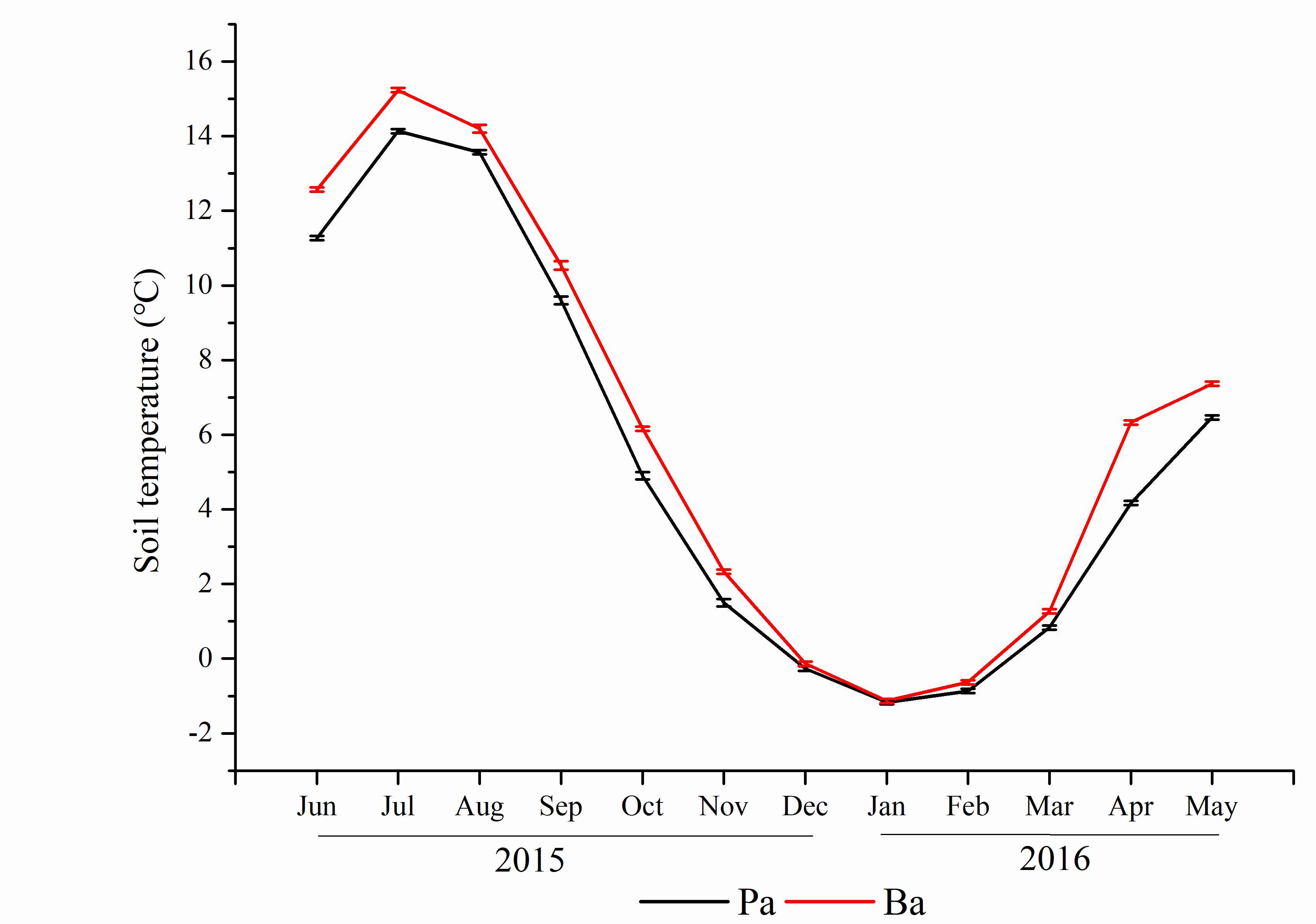


**Figure S1** Mean monthly soil temperature during the study period in *Betula albosinensis* (Ba) forest and *Picea asperata* Mast. (Pa) forest

(Figure S1 is created by Origin, version 9.0, 2012, OriginLab, Northampton, MA, USA; software available at: https://www.originlab.com/)

**Table S1** Basic soil properties across different seasons

| Forests | Seasons |  | pH | SOC (g·kg^−1^) | SWC (%) |
| --- | --- | --- | --- | --- | --- |
| Pa | Su |  | 5.9 | 65.5 | 39.5% |
|  | Au |  | 5.6 | 68.3 | 37.5% |
|  | Wi |  | 5.6 | 51.4 | 63.4% |
|  | Sp |  | 5.2 | 56.8 | 45.3% |
| Ba | Su |  | 6.1 | 69.8 | 35.9% |
|  | Au |  | 5.6 | 70.9 | 43.0% |
|  | Wi |  | 5.7 | 55.9 | 62.9% |
|  | Sp |  | 5.8 | 67.4 | 66.1% |

Pa: *Picea asperata* Mast.; Ba: *Betula albosinensis*; Sp: spring; Su: summer; Au: autumn; Wi: winter; SWC: soil water content; SOC: soil organic carbon;

**Table S2** Two-way ANOVA results for effects of vegetation type and season on LOC and their interactions with LOC

|  | **Forest (F)** | |  | **Season (Se)** | |  | F×Se | |
| --- | --- | --- | --- | --- | --- | --- | --- | --- |
|  | *F* | *p* |  | *F* | *p* |  | *F* | *p* |
| MBC | 1129.9 | < 0.01 |  | 7677.3 | < 0.01 |  | 151.3 | < 0.01 |
| EOC | 113.3 | < 0.01 |  | 60.5 | < 0.01 |  | 8.1 | < 0.01 |
| DOC | 702.6 | < 0.01 |  | 1005.2 | < 0.01 |  | 165.1 | < 0.01 |

Pa: *Picea asperata* Mast.; Ba: *Betula albosinensis*; MBC: microbial biomass carbon, EOC: easily oxidized organic carbon, DOC: dissolved organic carbon

(Data in Table S2 is analyzed by SPSS, version 20.0, 2011, SPSS Inc., Chicago, IL, USA; software available at: https://www.ibm.com/analytics/spss-statistics-software)

**Table S3** Two-way ANOVA results for effects of vegetation type and season on soil enzyme activities and their interactions with soil enzyme activities

|  | **Forest (F)** | |  | **Season (Se)** | |  | F×Se | |
| --- | --- | --- | --- | --- | --- | --- | --- | --- |
|  | *F* | *p* |  | *F* | *p* |  | *F* | *p* |
| CBH | 312.5 | < 0.01 |  | 319.3 | < 0.01 |  | 20.8 | < 0.01 |
| βG | 385.3 | < 0.01 |  | 382.7 | < 0.01 |  | 27.7 | < 0.01 |
| βX | 2016.2 | < 0.01 |  | 1720.5 | < 0.01 |  | 96.1 | < 0.01 |

Pa: *Picea asperata* Mast.; Ba: *Betula albosinensis*; CBH: cellobiohydrolase, βG: β-1,4-glucosidase, βX: β-1, 4-xylosidase

(Data in Table S3 is analyzed by SPSS, version 20.0, 2011, SPSS Inc., Chicago, IL, USA; software available at: https://www.ibm.com/analytics/spss-statistics-software)

Appendix I

Abbreviations and Nomenclatures

| **Name** | **Abbreviation** |
| --- | --- |
| labile organic carbon | LOC |
| microbial biomass carbon | MBC |
| easily oxidized organic carbon | EOC |
| dissolved organic carbon | DOC |
| *Betula albosinensis* | Ba |
| *Picea asperata* Mast. | Pa |
| cellobiohydrolase | CBH |
| β-1,4-glucosidase | βG |
| β-1,4-xylosidase | βX |
| soil water content | SWC |
| soil temperature | ST |
| 4-methylumbelliferyl | 4-MUB |
| operational taxonomic units | OTUs |
| ribosomal database project | RDP |
| redundancy analysis | RDA |
| *Acidobacteria* | Aci |
| *Proteobacteria* | Pro |
| *Bacteroidetes* | Bac |
| *Firmicutes* | Fir |
| *Actinobacteria* | Act |
| *Latescibacteria* | Lat |
| *Chloroflexi* | Chl |
| *Euryarchaeota* | Eur |
| *Basidiomycota* | Bas |
| *Ascomycota* | Asc |
